# Mr. Knowles' Case of Diarrhœa

**Published:** 1811-09

**Authors:** Edward Lloyd Knowles

**Affiliations:** Soham


*To the Editors of the Medical and Physical Journal.*

Gentlemen,

Any thing that leads to a successful treatment of diseases peculiar to mankind, especially when of an uncommon occurrence, probably, may not be unworthy of insertion in your excellent miscellany.

M. C———, a labouring-man's wife, of a debilitated habit of body, was affected, six weeks previous to my attendance, with diarrhœa, and being greatly emaciated from the violent evacuation, and the absorbents ceasing to perform their offices, symptoms of anasarca commenced, with a considerable enlargement of the lower extremities, nausea, vomiting, and excruciating pain accompanying every evacuation; so great was was the sinking that I was apprehensive of dissolution being not far off. The medical gentleman who had her first under his care, did not succeed in decreasing the peristaltic action and irritability in the intestinal canal; she gradually declined in strength until she was unable to make the smallest effort: at this time, which was six weeks from the commencement of the malady, I was desired to visit her, I found her in a very low state with nausea, vomiting, and great prostration of strength; the lower extremities cold and considerably enlarged, with a most violent pain accompanying every discharge *per anum*. I looked upon the predisposing cause to be some morbific matter producing irritation in the bowels, and that anasarca was only symptomatic of that affection; I was of opinion, previous to decreasing the action in the intestines, that a gentle aperient would be of utility; upon this ground I was determined to proceed. R. Pulv. Rhei Palmat. ʒss. Ol. Menth. pip. gtt. j. Tinct. Opii, gtt. xxv. Aq. pur. q. s. ft. haust. statim sumendus.

The opium in this instance was added to the aperient to obviate its stimulus upon the intestines, and with an intention of lessening excitement. Kino, Pulv. Cretæ, Opium, and several other remedies of the astringent and aromatic kind were administered, and after a long and tedious trial I succeeded to my satisfaction. My only motive now, was to direct my attention to the anasarcous affection of the lower extremities. I commenced with stimulating embrocations to augment the action of the decreased vessels, to absorb the accumulated lymph into the circulating system, and ordered her tonics, such as cinchona and the sulphate of steel, with proper nutritious regimen to support the *vis vitæ*. In two months, to my great astonishment, she was perfectly recovered from her tedious and severe indisposition. What is rather singular in this instance is, that the patient should lie so long without any intermission of the evacuation (having eight stools a day) without being exhausted, as she was naturally of a thin and delicate habit. Nature has in this case shewn a surprising effort to relieve herself of the morbific oppression, and it should give us a caution in other cases not to interfere or obstruct her progress too much when art is not absolutely necessary, but watch, discriminate, and assist her efforts.

I am, Gentlemen,

Your Humble Servant,

EDWARD LLOYD KNOWLES.

*Soham, June 5th,* 1811.

